# Drug-repurposing identified the combination of Trolox C and Cytisine for the treatment of type 2 diabetes

**DOI:** 10.1186/1479-5876-12-153

**Published:** 2014-05-31

**Authors:** Ling Jin, Jian Tu, Jianwei Jia, Wenbin An, Huanran Tan, Qinghua Cui, Zhixin Li

**Affiliations:** 1 Department of Pharmacology, Peking University Health Science Center, Beijing 100191, China; 2 State Key Laboratory of Natural and Biomimetic Drugs, Peking University Health Science Center, Beijing 100191, China; 3 Department of Electrocardiogram, Beijing Jishuitan Hospital, Beijing 100035, China; 4 Department of Biomedical Informatics, Peking University Health Science Center, Beijing 100191, China; 5 Department of Integrated Chinese and Western Medicine, Peking University Health Science Center, Beijing 100191, China

## Abstract

**Background:**

Drug-induced gene expression dataset (for example Connectivity Map, CMap) represent a valuable resource for drug-repurposing, a class of methods for identifying novel indications for approved drugs. Recently, CMap-based methods have successfully applied to identifying drugs for a number of diseases. However, currently few gene expression based methods are available for the repurposing of combined drugs. Increasing evidence has shown that the combination of drugs may valid for novel indications.

**Method:**

Here, for this purpose, we presented a simple CMap-based scoring system to predict novel indications for the combination of two drugs. We then confirmed the effectiveness of the predicted drug combination in an animal model of type 2 diabetes.

**Results:**

We applied the presented scoring system to type 2 diabetes and identified a candidate combination of two drugs, Trolox C and Cytisine. Finally, we confirmed that the predicted combined drugs are effective for the treatment of type 2 diabetes.

**Conclusion:**

The presented scoring system represents one novel method for drug repurposing, which would provide helps for greatly extended the space of drugs.

## Introduction

Drug repurposing or drug repositioning, which aims to find new therapeutic indications for approved drugs and experimental drugs that fail approval in their initial indication, has offered several advantages over traditional drug development including rescuing stalled pharmaceutical projects, finding therapies for neglected diseases and reducing the time, cost and risk of drug development [1,2]. During the past decade, a number of computational strategies for drug repurposing have been developed [1], including strategies based on the chemical similarity of drugs [3], similarity of drug side effects [4], molecular activity similarity [5], and shared molecular pathology [6]. Among these strategies, the method based on similarity of molecular activity generated from global gene expression profiling now emerges as a promising approach for drug repurposing [5]. Based on the premises of this technology, Connectivity Map (CMap) provides a data-driven and systematic approach for identifying associations among genes, drugs and disease. The publicity funded CMap reference catalogue initially contained profiles of 164 drugs and later expanded to around ~1400 FDA-approved small molecules. Furthermore, a number of CMap-based computational methods for drug repurposing have been developed and these methods have been successfully applied to discover drugs for a number of diseases [7-9]. For example, recently, Sirota et al. integrated a new gene expression database from 100 diseases and 164 drug compounds, yielding predicted novel therapeutic potentials for these drugs, such as antiulcer drug cimetidine as a candidate therapeutic in the treatment of lung adenocarcinoma [10].

In addition to individual drugs, now it is well known that drug combination may be used for novel indications [11-13]. More importantly, the drug combination will greatly extend the space of drugs but few computational methods are available [14]. For this purpose, here we presented a simple computational scoring system based on CMap and the deregulated gene profile of a given disease. We thus applied the presented scoring system to identify combinations of any two drugs in CMap for type 2 diabetes. Type 2 diabetes, a chronic metabolic disorder, has a strong effect on the quality of almost all aspects of life including health, social, and psychology. Generally, current therapeutic strategies for type 2 diabetes mainly involve insulin and four main classes of oral antidiabetic agents that stimulate pancreatic insulin secretion (sulphonylureas and rapid-acting secretagogues), reduce hepatic glucose production (biguanides), delay digestion and absorption of intestinal carbohydrate (a-glucosidase inhibitors) or improve insulin action (TZDs) [15]. However, each of the above agents is lack of effectiveness and suffers from a number of serious adverse effects. Due to complex molecular networks among biological systems and complicated interactions between genetic and environmental factors, new therapeutic agents or strategies are required for the treatment of type 2 diabetes. Finally, we identified a combination of Trolox C and Cytisine and confirmed that the predicted combination is effective for the treatment of type 2 diabetes.

## Materials and methods

### The CMap-based two-drug combination re-repurposing computational scoring system

**Figure 1:**
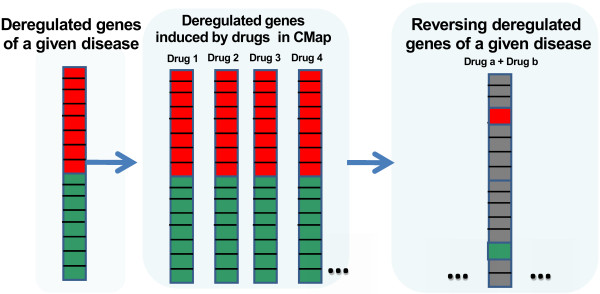
A scheme of the scoring system for computational drug-repurposing of combined drugs.

As shown in Figure 1, in this scoring system, we first identified the up/down regulated genes in a given disease, respectively. We then screened each drug in the CMap to identify drug induced up/down regulated genes. We next counted the number of genes deregulated in the disease that are reversed by each drug. Finally, we systematically evaluate the significance of any pair of drugs that reverse the disease genes. For example, for a given disease, drug one significantly reversed *m1* of the deregulated genes and induced the same deregulation of *n1* of the deregulated genes. These numbers for drug two is *m2* and *n2*. We take the product (*m1*-*n1*) × (*m2*-*n2*) as the score to evaluate the significance of the drug combination for the disease. A higher score represents a greater significance of the drug combination to reverse the deregulated genes in the disease.

### The deregulated genes

In this study, we took type 2 diabetes as an example to apply the presented scoring system. The up and down regulated genes in type 2 diabetes were obtained from the ArrayExpress database (http://www.ebi.ac.uk/arrayexpress/). As a result, we got 185 upregulated genes and 278 downregulated genes in type 2 diabetes, respectively (Additional file 1). We obtained the deregulated genes induced by drugs from the CMap database (http://www.broadinstitute.org/cmap/). The numbers of *m1*, *n1*, *m2*, and *n2* were calculated by in-house java and R programs.

### Animals and induction of diabetes mice

Three-week-old ICR male mice were purchased from Peking University Health Science Center (Beijing, China). Mice were accommodated under standard conditions (temp. 21 ± 2°C, 12:12 light–dark cycle, lights on at 7:00 a.m.) with food and water available *ad libitum*. After one-week rest, mice were treated four weeks on high fat diet (HFD). Experimental diabetes mice were induced afterwards by five daily injections of freshly prepared Streptozotocin (STZ) (40 mg/kg, i.p.) dissolved in 100 mM sodium acetate buffer (pH 4.5), while normal mice were injected with vehicle (sodium acetate buffer), all of them continued on the HFD. The HF/STZ model used in our experiment is that mice are fed with HF diet to induce insulin resistance followed by injection with STZ to induce partial pancreatic beta cell dysfunction. It is a popular T2DM model. Diabetes was assessed by monitoring blood glucose levels in fasted mice one week after STZ injection. The ones with blood glucose levels above 16.7 mM were considered diabetic and used in this study.

### Design of animal experiments

To explore the hypoglycemic activity of drugs, experiment was conducted on normal and diabetic mice, which were maintained on the same HFD. Normal mice were injected with saline (group NS), while diabetic mice were randomly assigned to one of the five groups including saline (group SS), insulin (group SI), Trolox C (group ST), Cytisine (group SC) and combination of Trolox C and Cytisine (group STC). Each group contains 10–14 mice. Cytisine (1 mg/kg, *i.p*., Sigma Aldrich) and Trolox C (50 mg/kg, *i.p*., Sigma Aldrich) were freshly diluted in phosphate buffered saline (pH 7.1) from stock solutions. Saline and drugs were administrated intraperitoneally every day (between 9 and 11 a.m.) for the entire four-week period. Body weight and fasting blood from mouse tails were measured before (pretreatment) drug administration and 1–4 weeks after drug or saline administration, respectively. An intraperitoneal glucose tolerance test (IPGTT) was conducted by intraperitoneal injection of a 20% glucose solution with the dose of 2 g kg^−1^ body weight. Both IPGTT and total area under the curve (AUC) were measured every two weeks. This study had been approved by the Animal Care Committee of the Peking University Health Science Center and all animal experiments were performed in compliance with the “Guidelines for Animal Experiment”.

### Statistical analysis

Data are shown as means ± standard error. Statistical analysis was performed by one-way ANOVA followed by a Tukey’s test and two-way ANOVA using Bonferroni’s test and t test. A p value less than 5% was considered significant (P < 0.05).

## Results

### Identifying candidate combinations of drugs for the treatment of type 2 diabetes

We applied the presented scoring system to scan the ~1400 drugs in CMap for type 2 diabetes. As a result, the predicted top ten candidate combinations of drugs are listed in Table 1. We next play to select one candidate combination for further animal experiments. One key rule of the selection is that none of the two combined drugs is reported to be associated diabetes. As a result, we selected the combination of Trolox C and Cytosine as the candidate drug combination for further animal experiment. One reason for selecting this combination is that none of the two drugs are reported to be associated with diabetes. Moreover, we manually mined information about the two drugs. Trolox C, one of vitamin E analogs, potentially offers significant advantages of anti-oxidation. With the ability of both aqueous and lipid solubility, of which aqueous solubility allows delivery to the target and lipid solubility enhances uptake by cell membranes, its antioxidant capacity is preserved and, in fact, appears to rival that of vitamin E. Investigations have suggested that Trolox C may permit myocardial salvage from acute myocardial ischemic injury [16], reduce the incidence of coronary artery disease [17], mitigate the toxic effects of several compounds in animal models [18], and protect muscle against disuse [19]. Cytisine, a natural plant alkaloid, has been marketed in Central and Eastern Europe as Tabex® for over 40 years for the clinical management of smoking cessation [20]. Numerous studies illustrate that Cytisine may have a more complex pharmacological function. As an acetylcholine agonist, Cytisine’s affinity for other nicotinic receptor subtypes, such as α4β4- or α6-containing subunits is or may be greater than or equal to its affinity for α4β2 subunits [21]. Based on our understanding towards Cytisine’s pharmacological function and its affinity to nicotinic receptors, this drug appears to have potential ability in treating other diseases, singly or in combination with other drugs. Therefore, here we select the combination of Trolox C and Cytisine for further animal experiment to evaluate its effectiveness for type 2 diabetes treatment.

**Table 1:** The predicted top ten combinations of drugs for the treatment of type 2 diabetes

**Drug 1**	**Drug 2**	**m1**	**n1**	**m2**	**n2**	**Score**
Trifluridine	Trolox C	22	8	61	24	518
Thiethylperazine	Trolox C	17	6	63	17	506
Cytisine	Trolox C	15	6	60	8	468
Cytisine	Ozagrel	23	8	48	20	420
Prenylamine	Thiethylperazine	17	1	42	16	416
Paclitaxel	Trolox C	12	4	61	10	408
Vinburnine	Trolox C	18	4	53	24	406
Ozagrel	Paclitaxel	22	7	47	20	405
Cytisine	Vancomycin	18	7	59	23	396
Valproic acid	Trifluridine	20	9	60	24	396

m1-the number of reversed genes by drug 1.

n1-the number of genes induced to the same deregulation as type 2 diabetes by drug 1.

M2-the number of reversed genes by drug 2.

n2-the number of genes induced to the same deregulation as type 2 diabetes by drug 2.

### Combination of Trolox C and Cytisine does not affect body weight in diabetic mice

**Figure 2:**
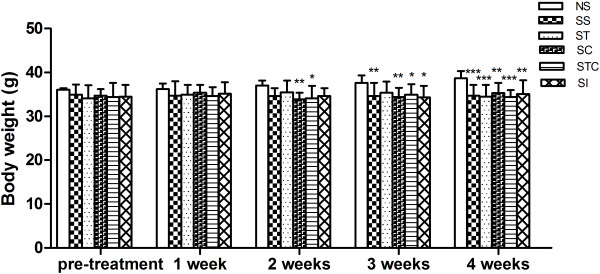
**Effectiveness of Trolox C and Cytisine on mice body weight.** Body weight of normal animals treated with saline solution (NS), STZ-induced animals treated with saline solution (SS), insulin (SI), Trolox C (ST), Cytisine (SC) and the combination of Trolox C and Cytisine (STC) were recorded (n = 5-10). The data are presented as means ± SEM. *p < 0.05, **p < 0.01, ***p < 0.001 vs NS.

Body weight was assessed on all six groups before drug administration and 1–4 weeks after drug or saline administration, respectively. There were no differences in base line body weight among groups at the beginning of experiment (pretreatment). Over the course of study, weight gain was observed in non-STZ-treated mice, while groups of STZ-treated mice showed no variation, this significant differences were recorded from 2w (Figure 2). The T2DM mice treated with the combination of Trolox C and Cytisine had similar body weight compared with the other groups of STZ-treated mice.

### Combination of Trolox C and Cytisine reverses diabetes in diabetic mice

**Figure 3:**
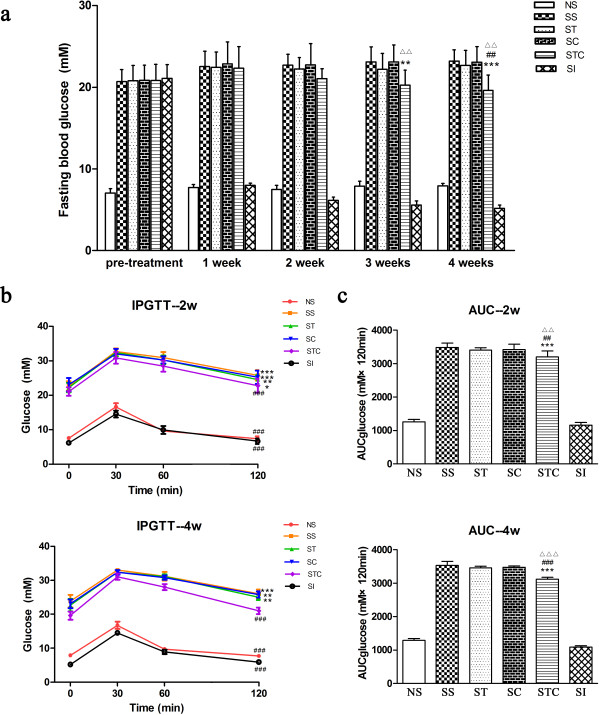
**Combination of Trolox C and Cytisine improved diabetes in diabetic mice. (a)** Fasting blood glucose concentration. **p < 0.01, ***p < 0.001 vs SS. ^##^P < 0.01 vs ST. ^△△^P < 0.01 vs SC. **(b)** Intraperitoneal glucose tolerance test of different groups at 2 weeks (upper) and 4 weeks (lower). *p < 0.05,**p < 0.01, ***p < 0.001 vs respective group at 0 min ^###^P < 0.001 vs SS at 120 min. **(c)** Total AUC calculated from glucose tolerance test data of 2 weeks(upper) and 4 weeks (lower). ***p < 0.001 vs SS. ^##^P < 0.01, ^###^P < 0.001 vs ST. ^△△^P < 0.01, ^△△△^P < 0.001 vs SC. n = 5-10. All data are presented as means ± SEM.

Consecutive five daily injection of STZ 40 mg/kg to normal mice significantly increased blood glucose levels from 7.06 ± 0.54 mM (NS, pretreatment) to 20.69 ± 1.47 mM (SS, pretreatment) after 7 days (P < 0.001), continued increasing to 22.53 ± 1.85 mM after14 days (SS, 1 week) (P < 0.001), and kept constantly high (SS, 2w, 3w, 4 w) (Figure 3a). Other STZ-treated groups of ST, SC and STC displayed similar trend. The hyperglycemic effect on mice showed the successful development of type 2 diabetes.Intraperitoneal injection of Trolox C to diabetic mice had no effect on fasting blood glucose levels (ST, 1w, 2w, 3w, 4w). Similar results were obtained with Cytisine injection (SC, 1w, 2w, 3w, 4w). While no effect of Cytisine or Trolox C alone, combination of the two drugs significantly reduced the level of STZ-induced hyperglycemia from 3w, as compared with saline- treated STZ mice (P < 0.01 in 3w, P < 0.001 in 4w). Co-treated mice showed less fasting glucose levels than Trolox C treated mice in 4 weeks (P < 0.01) and Cytisine treated mice in 3w and 4w (P < 0.01). Diabetic mice treated with insulin, as a positive control, showed obvious decreased glucose levels in all four weeks, as compared to other STZ-induced groups, and the reduction was significant in 4w compared NS mice (P < 0.05) (Figure 3a).Cytisine and Trolox C were also evaluated for intraperitoneal glucose tolerance (IPGTT) and total area under the curve (AUC) for glucose tolerance. As shown in Figure 3b, groups of SS, SC and ST displayed characteristic diabetic curves in 2w and 4w, where the blood glucose levels at 120 min were significantly higher than at 0 min (P < 0.01, P < 0.001). The SCT group showed similar difference in 2w (P < 0.05). The 120-min glucose levels of NS group and SI group in 2w and 4w, as well as SCT group in 4w were not different from those at 0 min. These three groups also showed significantly differences between SS group at 120 min. AS for the AUC, there was a reduced amount for mice of the STC group in 2w and 4w, as compared with the groups of SS, ST and SC (P < 0.01, P < 0.001). No difference was observed between groups of SS and ST, or groups of SS and SC (Figure 3c). All these demonstrated a partially alleviation of combination of Cytisine and Trolox C on glucose tolerance.

## Conclusions

Combinations of approved drugs could have novel uses. This strategy represents one class of novel methods for drug-repurposing. Here based on disease transcriptome and CMap-based drug-induced transcriptome, we presented a computational drug-repurposing scoring system to identify potential drug combinations for one given disease. We then applied this scoring system to type 2 diabetes, one severe metabolic disease. We identified the combination of Trolox C and Cytisine has the potential to treat type 2 diabetes, although none of them was reported to have the ability of treating type 2 diabetes. Finally, we confirmed that the predicted combination is effective to treat type 2 diabetes but none of them alone has the effectiveness. The presented scoring system provides an alternative solution to finding novel indications for approved drugs. Moreover, the identified combination of Trolox C and Cytisine provides a novel potential drug for type 2 diabetes.

## Discussion

In the present study, we presented a computational drug-repurposing scoring system to identify potential drug combinations for a given disease. Using this scoring system, we predicted drug combinations that could be used for treat type 2 diabetes. Finally, we select the combination of Trolox C and Cytisine for animal experiment. The result showed that the combination of Trolox C and Cytisine is effective for the treatment of type 2 diabetes but none of them are effective when being used alone. These results suggest that the presented method could provide helps in discovering drug combinations for a given disease.

Of course, limitations exist in the current study. One limitation is that the current computational method is not able to identify the optimal fractions of the two combined drugs. However, the fractions of the two drugs could play critical roles in their efficiency for treating disease. Second, some genomic information is not considered in this method. It is believed that some genomic information could be important for the drug-repurposing, for example, the importance of genes. The third limitation is that the changed expression fold of the deregulated genes is not considered in the current method. The current method is qualitative as it does not consider the extent of de-regulation and no significance was evaluated by statistical test. In addition, a random sampling technique would improve the prediction accuracy [22]. Therefore, in the future we would improve the CMap based computational methods for the identification of drug combination by considering the above limitations. However, it should be noted that the animal model of type 2 diabetes may not ideally mimic the procedure of human type 2 diabetes. Therefore, to further confirm and validate the hypoglycaemic effects of the combination of Trolox C and Cytisine in other type 2 diabetes animal models such as db/db mice, high-fat-diet-induced diabetic mice and rats, and OLETF rats will strengthen our observations that the combination of these two drugs may have some potential in treatment of human type 2 diabetes. Finally, although problems exist in the current method, we believed it present a simple and valuable alternative solution for drug-repurposing and would greatly extend the space of drugs.

## Competing interests

The authors declare that they have no competing interests.

## Authors’ contributions

QC, ZL, and HT designed the study. LJ carried out most of the animal experiments. JT carried out the computer programming. JJ and WA participated in the animal experiments. LJ, QC, and ZL wrote the manuscript. All authors read and approved the final manuscript.

## Supplementary Material

Additional file 1 The lists of upregulated genes and downregulated genes in type 2 diabetes. Click here for file
